# Factors associated with death from COVID-19 in traditional peoples and communities in Brazil

**DOI:** 10.1371/journal.pone.0327140

**Published:** 2025-07-10

**Authors:** Sebastião Bruno Taveira Silva, Audêncio Victor, Ana Raquel Ernesto Manuel Gotine, Dalva Maria de Assis, Marcelo Yoshito Wada, Greice Madeleine Ikeda do Carmo, Luciana Nogueira de Almeida Guimarães, Eucilene Alves Santana

**Affiliations:** 1 Ministry of Health, Secretariat of Health Surveillance and Environment, General Coordination of Immunopreventable Diseases Surveillance (CGVDI), Brasília, DF, Brazil; 2 School of Public Health, University of São Paulo (USP), Department of Public Health, São Paulo, SP, Brasil; 3 Department of Infectious Disease Epidemiology and International Health, London School of Hygiene Tropical Medicine, Keppel Street, London, United Kingdom; Faculty of Medicine of Jundiai, BRAZIL

## Abstract

**Introduction:**

COVID-19 has disproportionately impacted vulnerable populations, including traditional communities in Brazil, who face socioeconomic and health disparities, increasing the risk of severe outcomes. This study aims to identify factors associated with mortality among hospitalized COVID-19 patients from traditional communities in Brazil.

**Methods:**

This cross-sectional study analyzed data from the System of Epidemiological Surveillance of Influenza (SIVEP-Gripe) on hospitalizations for COVID-19 between 2021 and 2023. Individuals from traditional communities were included. The variables analyzed included demographic characteristics, clinical symptoms, comorbidities, and the need for hospital support. Logistic regressions were performed to assess associations with mortality, considering p < 0.05 significant.

**Results:**

Of the 7,101 cases analyzed, males showed a higher risk of death (OR = 1.39; 95% CI: 1.18–1.63). Among ethnic groups, blacks presented with an OR = 2.92 (95% CI: 1.68–5.08) and indigenous, OR = 2.25 (95% CI: 1.73–2.94). Older age increased the risk, with OR = 6.57 (95% CI: 2.16–28.5) for ages 60–79 and OR = 12.8 (95% CI: 4.18–55.8) for ≥80 years. Dyspnea (OR = 2.16; 95% CI: 1.77–2.65) and low saturation (OR = 2.13; 95% CI: 1.78–2.55) were associated with death, while loss of taste was protective (OR = 0.62; 95% CI: 0.51–0.75). Immunosuppression (OR = 2.14; 95% CI: 1.23–3.79) and chronic renal disease (OR = 1.64; 95% CI: 1.10–2.46) increased the risk. Patients on invasive ventilation had the highest risk of death (OR = 19.4; 95% CI: 15.2–25.0), followed by non-invasive ventilation (OR = 2.65; 95% CI: 2.18–3.23) and ICU (OR = 2.15; 95% CI: 1.85–2.49).

**Conclusion:**

Risk factors for mortality among hospitalized patients from traditional communities include male sex, older age, race/color, severe respiratory symptoms, comorbidities, and the need for invasive ventilation. These findings reinforce the importance of targeted health strategies to reduce the risk of mortality in these vulnerable populations.

## Introduction

COVID-19, caused by the coronavirus SARS-CoV-2, emerged in Wuhan, China, in December 2019 and was quickly recognized by the World Health Organization (WHO) as a public health emergency of international concern by January 2020 [1]. The rapid global spread of the virus led WHO to declare a global pandemic by March 2020 due to its high transmissibility and significant impact on public health systems worldwide [1–4].

In Brazil, the pandemic has disproportionately affected Indigenous and other traditional communities, highlighting existing vulnerabilities. The first recorded case of COVID-19 in an Indigenous person was in June 2020. Within a month, the numbers rose sharply, indicating a severe impact on these communities [5]. Epidemiological data from April 2024 indicate a decline in case numbers and deaths, likely attributed to increased vaccination coverage and targeted public health interventions. However, the mortality rate from severe acute respiratory syndrome caused by COVID-19 remained highest among Indigenous populations in the states of Amazonas, Rio Grande do Sul, and Rio Grande do Norte [6]. Traditional peoples and communities in Brazil include Indigenous peoples, quilombolas (Afro-Brazilian communities), riverine populations, extractivist communities, and others recognized under Brazilian Federal Laws No. 11.326/2006 and No. 14.021/2020.

Studies have consistently shown that traditional communities are at greater risk during infectious disease outbreaks, with the COVID-19 pandemic exacerbating existing health disparities. The mortality rate among Indigenous populations was up to 6.5 times higher than the national average at the pandemic’s peak in July 2020, underscoring the urgent need for targeted health interventions [2,7,8]. Critical barriers such as inadequate access to potable water, basic sanitation, and vaccination have hindered effective disease management in these populations. Additionally, cultural practices and strong connections with the environment have influenced disease transmission and the pandemic’s broader social impacts [9]. Further research into demographic and environmental factors reveals that advanced age, male sex, presence of comorbidities, and residing in areas of low social development significantly correlate with COVID-19 severity and mortality [10–13]. For instance, studies from Amapá have highlighted that most COVID-19 deaths among Indigenous people occurred in older individuals with cardiovascular comorbidities, indicating a heightened vulnerability within this group [14].

The legislative response, marked by the enactment of Law No. 14.021 on July 7, 2020, established specific epidemiological surveillance and preventive measures for COVID-19 among traditional communities. In March 2021, Brazil’s health surveillance systems, e-SUS Notifica and Sivep-Gripe, began categorizing these communities following a Supreme Federal Court mandate, enhancing case identification and [15]. This study aims to analyze factors associated with COVID-19 mortality in traditional communities, identifying key determinants to inform public health policies that reduce disparities and improve health outcomes.

## Methods

### Study design and population

This cross-sectional study was conducted with individuals hospitalized with severe acute respiratory syndrome (SARS) due to COVID-19, belonging to traditional peoples and communities in Brazil. The study population included all hospitalized individuals identified as traditional peoples and communities across all states of Brazil, with a laboratory-confirmed diagnosis of COVID-19 registered in the influenza epidemiological surveillance information system (SIVEP-Gripe), between 2021 and 2023. COVID-19 diagnosis was primarily confirmed by real-time polymerase chain reaction (RT-PCR), as recorded in the database. The temporal and population cut-offs were defined based on the inclusion of the variable that identifies traditional peoples and communities in SIVEP-Gripe, allowing specific analysis of these vulnerable populations [16].

### Data source

The data were extracted from SIVEP-Gripe, maintained by the Brazilian Ministry of Health, which covers notifications of hospitalizations for SARS across the national territory [17]. This system is crucial for the epidemiological surveillance of respiratory diseases, including COVID-19, and provides detailed information about demographic, clinical characteristics, and patient outcomes, and has been widely used in epidemiological studies for its scope and reliability in collecting data on respiratory diseases in the country [18,19]. Exclusively secondary and aggregate public domain data were used. Therefore, informed consent and approval by a Research Ethics Committee are waived, according to Resolution No. 466/2012 of the National Commission on Research Ethics of the National Health Council in Brazil.

### Study variables

The study variables were organized into different categories. First, sociodemographic characteristics included variables such as sex (male, female), race/color (white, black, yellow, brown, indigenous), age group (<1 year, 1–4 years, 5–11 years, 12–19 years, 20–59 years, 60–79 years, and ≥80 years), education (no education, elementary 1st cycle, elementary 2nd cycle, high school, higher education), and area of residence (urban, peri-urban, rural). Clinical symptoms were recorded as binary variables (yes/no) and included: fever, cough, sore throat, dyspnea, respiratory distress, oxygen saturation <95%, diarrhea, vomiting, abdominal pain, fatigue, loss of smell, and loss of taste. Comorbidities were also analyzed as binary variables (yes/no), including diabetes, obesity, chronic neurological disease, asthma, chronic lung disease, chronic cardiovascular disease, immunosuppression, chronic kidney disease, chronic liver disease, chronic hematological disease, Down syndrome, and puerperal condition. Finally, hospital support-related variables were analyzed: presence of general risk factors (yes/no), COVID-19 vaccination status (yes/no), type of ventilatory support (none, non-invasive, invasive), and ICU admission (yes/no).

### Outcome

The study outcome was the progression of hospitalization, categorized as death or hospital discharge. This variable was used to compare the characteristics of individuals who survived hospitalization and those who progressed to death.

### Statistical analysis

Initially, a descriptive analysis of the categorical variables was conducted, presenting absolute and relative frequencies for the discharge and death groups. The Pearson chi-square test was employed to evaluate associations between categorical variables and the outcome. To identify risk factors associated with death, a bivariate and multivariate logistic regression analysis was performed [20]. Variables with a p-value < 0.20 in the bivariate analysis were included in the multivariate model, using the stepwise selection method [21]. The multivariate logistic regression allowed for adjusting the model for potential confounding factors, quantifying the magnitude of association with the outcome through odds ratios (OR) and 95% confidence intervals (CI 95%). All analyses were conducted using R software, version 4.3.0.

## Results

### Sociodemographic Characteristics and Mortality

The analysis included 7,101 cases of SARS due to COVID-19 among individuals from traditional peoples and communities in Brazil, registered in SIVEP-Gripe between 2021 and 2023. As shown in **Tables 1 and 2**, the majority of patients self-identified as indigenous (59.13%), followed by “others not identified” (30.28%), with other communities, such as quilombolas and riverine, representing smaller percentages, all below 2%. Regarding sex, 38% of men progressed to death, while among women the rate was 32% (p < 0.001). The majority of indigenous individuals were discharged (64%), and 36% progressed to death (p < 0.001), while the white population had the lowest death rate, at 28% (p < 0.001). Education was also a relevant factor; patients with no schooling had a death rate of 49%, while those with high school education had a rate of 28% (p < 0.001). Individuals from rural areas had a slightly higher death rate (37%) compared to those from urban areas (34%) (p = 0.025).

**Table 1:** Characterization of traditional peoples and communities with SARS due to COVID-19, Brazil, 2021 to 2023. (N = 7,101).

Type of traditional peoples and communities	n	(%)
Indigenous	4.199	59,13
Others not identified	2.150	30,28
Riverine	129	1,82
Quilombola	128	1,80
Stateless persons	91	1,28
Encamped	83	1,17
Salaried rural workers	76	1,07
Settlers	75	1,06
Other group	170	2,39

**Table 2:** Distribution of sociodemographic characteristics, clinical symptoms, comorbidities, and hospital support factors among patients from traditional peoples and communities hospitalized with SARS due to COVID-19 in Brazil, 2021-2023 (N = 7,101).

Variable	Total (n)	Recovery, N = 4,606 ^1^	Death, N = 2,495 ^1^	p-value ^2^
**Sex**				<0.001
Male	3924	2,447 (62%)	1,477 (38%)	
Female	3177	2,159 (68%)	1,018 (32%)	
**Race/Color**				<0.001
Indigenous	4,156	2,674 (64%)	1,482 (36%)	
Brown	1,432	879 (61%)	553 (39%)	
White	1,171	838 (72%)	333 (28%)	
Black	163	93 (57%)	70 (43%)	
Yellow	82	54 (66%)	28 (34%)	
**Age group**				<0.001
<1 year	145	123 (85%)	22 (15%)	
1 to 4 years	183	148 (81%)	35 (19%)	
5 to 11 years	216	184 (85%)	32 (15%)	
12 to 19 years	167	147 (88%)	20 (12%)	
20 to 59 years	3,075	2,326 (76%)	749 (24%)	
60 to 79 years	2,297	1,252 (55%)	1,045 (45%)	
80 years or older	1,018	426 (42%)	592 (58%)	
**Education**				<0.001
No education	700	358 (51%)	342 (49%)	
Primary education (1st cycle)	1,029	622 (60%)	407 (40%)	
Primary education (2nd cycle)	580	367 (63%)	213 (37%)	
High school	603	434 (72%)	169 (28%)	
Higher education	189	132 (70%)	57 (30%)	
**Area**				0.025
Urban	3,875	2,543 (66%)	1,332 (34%)	
Rural	2,405	1,518 (63%)	887 (37%)	
Peri-urban	55	42 (76%)	13 (24%)	
**Signs and symptoms**				0.055
Yes	6,856	4,433 (65%)	2,423 (35%)	
No	245	173 (71%)	72 (29%)	
**Fever**				0.196
Yes	4,506	2,926 (65%)	1,580 (35%)	
No	1,593	1,063 (67%)	530 (33%)	
**Cough**				0.670
Yes	5,375	3,516 (65%)	1,859 (35%)	
No	949	614 (65%)	335 (35%)	
**Sore throat**				0.891
No	3,335	2,173 (65%)	1,162 (35%)	
Yes	1,746	1,141 (65%)	605 (35%)	
**Dyspnea**				<0.001
Yes	5,035	3,014 (60%)	2,021 (40%)	
No	1,109	907 (82%)	202 (18%)	
**Respiratory distress**				<0.001
Yes	4,440	2,714 (61%)	1,726 (39%)	
No	1,514	1,103 (73%)	411 (27%)	
**Oxygen saturation**				<0.001
O2 < 95%	4,564	2,686 (59%)	1,878 (41%)	
O2 ≥ 95%	1,411	1,131 (80%)	280 (20%)	
**Diarrhea**				0.008
No	3,905	2,507 (64%)	1,398 (36%)	
Yes	954	656 (69%)	298 (31%)	
**Vomiting**				0.061
No	4,111	2,653 (65%)	1,458 (35%)	
Yes	680	464 (68%)	216 (32%)	
**Abdominal pain**				0.372
No	3,528	2,341 (66%)	1,187 (34%)	
Yes	564	385 (68%)	179 (32%)	
**Fatigue**				0.592
No	2,496	1,674 (67%)	822 (33%)	
Yes	1,908	1,265 (66%)	643 (34%)	
**Loss of smell**				<0.001
No	3,373	2,219 (66%)	1,154 (34%)	
Yes	722	522 (72%)	200 (28%)	
**Loss of taste**				<0.001
No	3,351	2,201 (66%)	1,150 (34%)	
Yes	735	538 (73%)	197 (27%)	
**Comorbidity**				<0.001
No	4,280	3,004 (70%)	1,276 (30%)	
Yes	2,821	1,602 (57%)	1,219 (43%)	
**Diabetes**				0.007
Yes	1,307	713 (55%)	594 (45%)	
No	1,230	736 (60%)	494 (40%)	
**Obesity**				0.950
No	1,636	935 (57%)	701 (43%)	
Yes	465	265 (57%)	200 (43%)	
**Chronic neurological disease**				0.007
No	1,891	1,087 (57%)	804 (43%)	
Yes	255	169 (66%)	86 (34%)	
**Asthma**				0.041
No	1,850	1,045 (56%)	805 (44%)	
Yes	149	97 (65%)	52 (35%)	
**Chronic lung disease**				0.028
No	1,838	1,057 (58%)	781 (42%)	
Yes	189	93 (49%)	96 (51%)	
**Chronic cardiovascular disease**				0.008
Yes	1,608	892 (55%)	716 (45%)	
No	1,097	665 (61%)	432 (39%)	
**Immunosuppression**				0.004
No	1,873	1,078 (58%)	795 (42%)	
Yes	96	41 (43%)	55 (57%)	
**Chronic renal disease**				<0.001
No	1,816	1,043 (57%)	773 (43%)	
Yes	210	93 (44%)	117 (56%)	
**Chronic liver disease**				0.001
No	1,915	1,098 (57%)	817 (43%)	
Yes	52	18 (35%)	34 (65%)	
**Chronic hematological disease**				0.714
No	1,942	1,105 (57%)	837 (43%)	
Yes	35	21 (60%)	14 (40%)	
**Down syndrome**				0.274
No	1,958	1,121 (57%)	837 (43%)	
Yes	18	8 (44%)	10 (56%)	
**Postpartum**				0.003
No	1,901	1,074 (56%)	827 (44%)	
Yes	55	42 (76%)	13 (24%)	
**COVID-19 Vaccine**				0.044
No	1,923	1,266 (66%)	657 (34%)	
Yes	1,470	1,016 (69%)	454 (31%)	
**Ventilatory support**				<0.001
Non-invasive	3,372	2,415 (72%)	957 (28%)	
None	1,466	1,243 (85%)	223 (15%)	
Invasive	1,165	225 (19%)	940 (81%)	
**ICU**				<0.001
No	3,986	3,111 (78%)	875 (22%)	
Yes	1,928	771 (40%)	1,157 (60%)	

^1^n (%); ^2^Pearson’s Chi-squared test.

### Clinical conditions, comorbidities, and hospital support

The presence of severe respiratory symptoms, such as dyspnea (40% death) and respiratory distress (39% death), was strongly associated with a higher risk of death (p < 0.001). Patients with oxygen saturation below 95% had a death rate of 41%, while those with normal saturation had a rate of 20% (p < 0.001). Loss of smell and taste were associated with lower death rates, suggesting a possible association with less severe COVID-19 cases (p < 0.001). Among comorbidities, patients with diabetes (45%, p = 0.007), chronic cardiovascular disease (45%, p = 0.008), and immunosuppression (57%, p = 0.004) had higher risks of death. Chronic renal and liver diseases also showed a strong association with death, with rates of 56% and 65%, respectively (p < 0.001 and p = 0.001). Patients who required invasive ventilation exhibited the highest death rate, reaching 81% (p < 0.001), indicating the severity of cases that require this support. Furthermore, the need for ICU admission was also a significant risk factor, with 60% of those admitted to the ICU progressing to death (p < 0.001), **Table 2**.

### Factors associated with mortality

**Fig 1:**
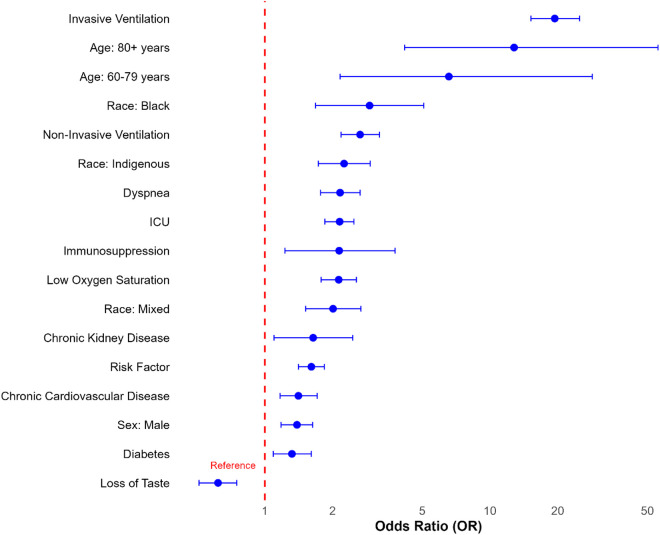
Significant risk factors associated with COVID-19 mortality in traditional peoples and communities with SARS due to COVID-19 in Brazil, 2020 to 2023. Odds ratios (OR) and 95% confidence intervals are displayed for key clinical and sociodemographic variables. A red dashed line indicates the reference value (OR = 1). Variables with OR > 1 indicate increased risk of death.

As shown in **Table 3 and Fig 1**, the multivariate analysis revealed that, among individuals from traditional communities hospitalised with COVID-19, male sex was associated with an increased risk of mortality with an OR = 1.39 (95% CI: 1.18–1.63). Regarding race/color, self-declared black individuals had an OR = 2.92 (95% CI: 1.68–5.08) and indigenous an OR = 2.25 (95% CI: 1.73–2.94) for the risk of death. Advanced age was shown to be a substantial risk factor, with an OR = 6.57 (95% CI: 2.16–28.5) for those between 60 and 79 years and an OR = 12.8 (95% CI: 4.18–55.8) for individuals aged 80 years or more. Individuals without education also showed an increased risk of mortality, with an OR = 1.32 (95% CI: 0.89–1.96), although this association was not significant in the adjusted model. Among signs and symptoms, dyspnea had an OR = 2.16 (95% CI: 1.77–2.65) and low oxygen saturation presented an OR = 2.13 (95% CI: 1.78–2.55), both strongly associated with the risk of a fatal outcome. In contrast, loss of taste was shown to be a protective factor with an OR = 0.62 (95% CI: 0.51–0.75), indicating a possible association with less severe COVID-19 cases. Comorbidities were also significant determinants of mortality risk, with immunosuppression associated with an OR = 2.14 (95% CI: 1.23–3.79) and chronic renal disease with an OR = 1.64 (95% CI: 1.10–2.46). In terms of hospital support, patients requiring invasive ventilation had the highest risk of death, with an OR = 19.4 (95% CI: 15.2–25.0). Those using non-invasive ventilation had an OR = 2.65 (95% CI: 2.18–3.23), and ICU admission was associated with an OR = 2.15 (95% CI: 1.85–2.49).

**Table 3:** Factors associated with death in traditional peoples and communities with SARS due to COVID-19 in Brazil, 2020 to 2023.

Variables	Bivariate logistic regression			Multivariate logistic regression*		
	OR	IC95%	p-value	OR	IC95%	p-value
**Sexo**						
**Sex**	ref	–	–	ref	–	–
Female	1,28	1,16−1,41	<0,001	1,39	1,18− 1,63	<0,001
Male						
**Race/Color**	ref	–	–			
White	1,89	1,35 −2,64	<0,001	2,92	1,68− 5,08	<0,001
Black	1,58	1,34 − 1,87	<0,001	2,01	1,52− 2,67	<0,001
Brown	1,39	1,21 − 1,61	<0,001	2,25	1,73− 2,94	<0,001
Indigenous	1,3	0,80 − 2,08	0,3	1,61	0,57− 4,24	0,3
Asian						
**Age Group**	ref	–	–			
< 1 year	1,32	0,74−2,40	0,30	0,77	0,17− 4,13	0,7
1 to 4 years	0,97	0,54−1,77	>0,9	0,3	0,05− 1,74	0,2
5 to 11 years	0,76	0,39−1,46	0,40	0,76	0,19− 3,80	0,7
12 to 19 years	1,8	1,16−2,93	0,012	2,41	0,79− 10,5	0,2
20 to 59 years	4,67	3,01-7,59	<0,001	6,57	2,16- 28,5	0,003
60 to 79 years	7,77	4,95−12,70	<0,001	12,8	4,18- 55,8	<0,001
80 years or more						
**Education**	ref	–	–			
Higher education	2,21	1,58, 3,14	<0,001	1,32	0,89− 1,96	0,2
No schooling	1,52	1,09, 2,13	0,015	1,18	0,82− 1,71	0,4
Primary (1st cycle)	1,34	0,95, 1,92	0,10	1,16	0,80− 1,71	0,4
Primary (2nd cycle)	0,9	0,63, 1,30	0,6	0,91	0,62− 1,34	0,6
Secondary						
**Area**	ref	–	–			
Urban	1,12	1,00-1,24	0,043	0,84	0,70− 1,00	0,052
Rural	0,59	0,30−1,07	0,1	0,23	0,08− 0,55	0,002
Periurban						
**Dyspnea**	ref	–	–			
No	3,01	2,55−3,54	<0.001	2,16	1,77− 2,65	<0,001
Yes						
**Oxygen Saturation**	ref	–	–			
Normal	2,82	2,44−3,26	<0.001	2,13	1,78− 2,55	<0,001
Low						
**Loss of Taste**	ref	–	–			
No	0,7	0,58−0,83	<0.001	0,62	0,51− 0,75	<0,001
Yes						
**Diabetes**	ref	–	–			
No	1,24	1,06−1,45	0,008	1,32	1,09− 1,61	0,005
Yes						
**Chronic Cardiovascular Disease**	ref	–	–			
Yes	1,23	1,05−1,44	0,008	1,41	1,17 − 1,71	<0,001
**Immunosuppression**						
No	ref	–	–			
Yes	1,81	1,20−2,75	0,004	2,14	1,23 − 3,79	0,007
**Chronic Kidney Disease**						
No	ref	–	–			
Yes	1,69	1,27−2,26	<0.001	1,64	1,10−2,46	0,017
**Ventilatory Support**						
Not used	ref	–	–			
Non-invasive	2,21	1,88 − 2,60	<0.001	2,65	2,18 − 3,23	<0,001
Invasive	23,3	19,0 - 28,6	<0.001	19,4	15,2 - 25,0	<0,001
**ICU**						
No	ref	–	–			
Yes	5,33	4,74−6,00	<0.001	2,15	1,85 − 2,49	<0,001

CI: 95% Confidence Interval; OR:

## Discussion

The specific vulnerabilities of Brazil’s traditional communities to COVID-19 highlight critical aspects of pandemic response and mortality risks within these populations. In this study, we observed that a significant majority of patients were Indigenous (59.13%), followed by a diverse group of “other non-identified” (30.28%), with smaller representations from quilombolas and riverine communities, all under 2%. These findings align with the national epidemiological data indicating that traditional communities, particularly Indigenous peoples, face disproportionately higher risks of COVID-19-related mortality [15].

Our results corroborate earlier studies indicating elevated mortality risks among non-white populations, with Black and Indigenous individuals experiencing significantly higher mortality rates compared to the national average [22]. This disparity is not merely statistical but reflects deep-rooted social and structural mechanisms, including systemic racism, geographical isolation, inadequate access to healthcare, and historical marginalization. For example, research in the Brazilian Amazon found that Indigenous populations had a 110% higher mortality rate from COVID-19 than the national average, emphasizing the impact of systemic health disparities [23,24]. Black individuals have similarly been independently associated with higher COVID-19 mortality [25]. Comparable trends in the United States revealed that, in Chicago, Black residents accounted for 70% of COVID-19 deaths despite representing only 29% of the population. In Michigan, Black individuals made up 14% of the population but accounted for 30% of cases and 40% of deaths, likely due to barriers in healthcare access and socioeconomic conditions [8,26]. Our findings likely reflect persistent inequalities in access to timely healthcare, the higher prevalence of pre-existing conditions, and delays in receiving adequate hospital support among traditional communities. Moreover, structural racism and geographic isolation may further exacerbate these risks.

Gender differences were also evident, with male patients showing a higher mortality risk. This pattern, consistent with global data, may be explained by biological and behavioural factors. Biologically, men tend to have weaker immune responses and a higher prevalence of comorbidities such as hypertension and cardiovascular disease, which aggravate COVID-19 severity. Hormonal differences, particularly the protective effect of estrogen in women, may also influence immune modulation and reduce the severity of respiratory infections [27,28].

Advanced age was another substantial risk factor, with the highest mortality rates observed among individuals aged 80 and above, mirroring other findings where mortality increases with age, particularly among Indigenous populations [2,10,13]. In a Rio de Janeiro cohort, age brackets associated with mortality were 70–79 (OR = 1.5), 80–89 (OR = 3.1), and 90–99 years (OR = 5.1) [12]. Regarding clinical presentation, symptoms like dyspnea and low oxygen saturation were closely associated with fatal outcomes. For instance, mortality among patients reporting dyspnea in Pará increased by 676.6% [29]; with other symptoms such as nasal congestion and nausea showing similar trends [30]. Comorbidities were significant mortality predictors, particularly immunosuppression. A study in Amapá found that Indigenous patients with cardiovascular comorbidities had a 4.01 times higher mortality risk from COVID-19 than those without [14]. Meta-analyses further support the association between cardiovascular comorbidities, including hypertension, and increased mortality risk in the general population [11].

Hospital support factors also played a critical role. Patients requiring invasive ventilation faced the highest mortality risk, while non-invasive ventilation and ICU admission also correlated with higher mortality [31]. These findings underscore the importance of vaccination, which has been shown to reduce severe cases and critical outcomes in COVID-19 [32]. Our study adds to the literature by emphasizing how these clinical and demographic risk factors intersect with the social realities of traditional communities. This intersection likely amplifies vulnerability and highlights the need for equity-focused public health strategies.

This study is subject to several limitations. First, we relied on secondary data from the SIVEP-Gripe surveillance system, which may present inconsistencies and underreporting, particularly in remote areas. Missing data were present for several variables, and only complete cases were included in multivariable models, which may have led to selection bias. Second, the classification of race/ethnicity and community type is based on administrative records and may not capture the full sociocultural identity of the patients. Third, we were unable to evaluate the timing of access to care, availability of oxygen, or hospital-level differences, all relevant factors in explaining mortality variation. Despite these limitations, our study has several strengths. We utilized a large, nationwide database that captured severe cases across all Brazilian regions and applied rigorous statistical analysis. To our knowledge, this is one of the few studies focusing on COVID-19 mortality among traditional peoples and communities, a group that has historically been underrepresented in health research. By analyzing multiple factors (clinical, demographic, support-related), we were able to build a more comprehensive profile of mortality risks. Future research should explore longitudinal outcomes, evaluate the effectiveness of vaccination campaigns in these groups, and incorporate qualitative approaches to understand social barriers to care better. Monitoring mortality and morbidity among traditional communities must remain a priority in future epidemic preparedness agendas.

## Conclusion

This study demonstrated that factors such as male sex, advanced age, race/color, severe respiratory symptoms, comorbidities, and the need for invasive ventilation are associated with mortality among hospitalized COVID-19 patients from traditional communities in Brazil. These groups face additional vulnerabilities due to socioeconomic inequalities and barriers to healthcare access. The findings underscore the need for targeted public policies focused on prevention, access to intensive care, and early treatment, especially for those with chronic conditions. Specific strategies can reduce mortality and ensure more equitable care.

## Supporting information

S1 Data Anonymized dataset and R script used for analysis of factors associated with COVID-19 mortality in traditional peoples and communities in Brazil (2021–2023). (RDATA)
