# Correction: Factors associated with death from COVID-19 in traditional peoples and communities in Brazil

**DOI:** 10.1371/journal.pone.0344147

**Published:** 2026-03-02

**Authors:** Sebastião Bruno Taveira Silva, Audêncio Victor, Ana Raquel Ernesto Manuel Gotine, Dalva Maria de Assis, Marcelo Yoshito Wada, Greice Madeleine Ikeda do Carmo, Luciana Nogueira de Almeida Guimarães, Eucilene Alves Santana

Table 3 was uploaded incorrectly. Please see the correct Table 3 below.

**Table 3:** Factors associated with death in traditional peoples and communities with SARS due to COVID-19 in Brazil, 2020 to 2023.

Variables	Bivariate logistic regression			Multivariate logistic regression *		
	OR	IC95%	p-value	OR	IC95%	p-value
**Sex**						
Female	ref	–	–	–	–	–
Male	1,28	1,16−1,41	<0,001	1,39	1,18− 1,63	<0,001
**Race/Color**						
White	ref	–	–			
Black	1,89	1,35 −2,64	<0,001	2,92	1,68− 5,08	<0,001
Brown	1,58	1,34 − 1,87	<0,001	2,01	1,52− 2,67	<0,001
Indigenous	1,39	1,21 − 1,61	<0,001	2,25	1,73− 2,94	<0,001
Yellow/Asian	1,3	0,80 − 2,08	0,3	1,61	0,57− 4,24	0,3
**Age Group**						
< 1 year	ref	–	–	–	–	–
1 to 4 years	1,32	0,74−2,40	0,30	0,77	0,17− 4,13	0,7
5 to 11 years	0,97	0,54−1,77	>0,9	0,3	0,05− 1,74	0,2
12 to 19 years	0,76	0,39−1,46	0,40	0,76	0,19− 3,80	0,7
20 to 59 years	1,8	1,16−2,93	0,012	2,41	0,79− 10,5	0,2
60 to 79 years	4,67	3,01-7,59	<0,001	6,57	2,16- 28,5	0,003
80 years or more	7,77	4,95−12,70	<0,001	12,8	4,18- 55,8	<0,001
**Education**						
Higher education	ref	–	–	–	–	–
No schooling	2,21	1,58, 3,14	<0,001	1,32	0,89− 1,96	0,2
Primary (1st cycle)	1,52	1,09, 2,13	0,015	1,18	0,82− 1,71	0,4
Primary (2nd cycle)	1,34	0,95, 1,92	0,10	1,16	0,80− 1,71	0,4
Secondary	0,9	0,63, 1,30	0,6	0,91	0,62− 1,34	0,6
**Area**						
Urban	ref	–	–			
Rural	1,12	1,00-1,24	0,043	0,84	0,70− 1,00	0,052
Periurban	0,59	0,30−1,07	0,1	0,23	0,08− 0,55	0,002
**Dyspnea**						
No	ref	–	–			
Yes	3,01	2,55−3,54	<0.001	2,16	1,77− 2,65	<0,001
**Oxygen Saturation**						
Normal	ref	–	–			
Low	2,82	2,44−3,26	<0.001	2,13	1,78− 2,55	<0,001
**Loss of Taste**						
No	ref	–	–			
Yes	0,7	0,58−0,83	<0.001	0,62	0,51− 0,75	<0,001
**Diabetes**						
No	ref	–	–			
Yes	1,24	1,06−1,45	0,008	1,32	1,09− 1,61	0,005
**Chronic Cardiovascular Disease**						
No	ref	–	–			
Yes	1,23	1,05−1,44	0,008	1,41	1,17 − 1,71	<0,001
**Immunosuppression**						
No	ref	–	–			
Yes	1,81	1,20−2,75	0,004	2,14	1,23 − 3,79	0,007
**Chronic Kidney Disease**						
No	ref	–	–			
Yes	1,69	1,27−2,26	<0.001	1,64	1,10−2,46	0,017
**Ventilatory Support**						
Not used	ref	–	–			
Non-invasive	2,21	1,88 − 2,60	<0.001	2,65	2,18 − 3,23	<0,001
Invasive	23,3	19,0 - 28,6	<0.001	19,4	15,2 - 25,0	<0,001
**ICU**						
No	ref	–	–			
Yes	5,33	4,74−6,00	<0.001	2,15	1,85 − 2,49	<0,001

*CI: 95% Confidence Interval; OR: Odds Ratio; Ref: Reference.*
